# Concurrent Conflicts—the Great War and the 1918 Influenza Pandemic

**DOI:** 10.3201/eid2410.AC2410

**Published:** 2018-10

**Authors:** Terence Chorba, Byron Breedlove

**Affiliations:** Author affiliation: Centers for Disease Control and Prevention, Atlanta, Georgia, USA

**Keywords:** art science connection, emerging infectious diseases, art and medicine, about the cover, John Singer Sargent, Interior of a Hospital Tent, Concurrent Conflicts, the Great War and the 1918 Influenza Pandemic, 1918 Influenza Pandemic, World War I, the Spanish flu, influenza, H1N1, public health, viruses

**Figure:**
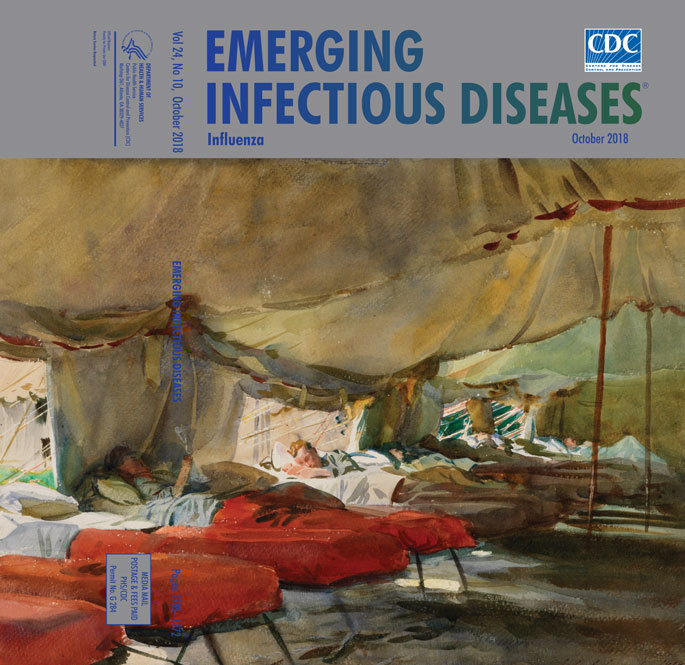
**John Singer Sargent (1856–1925), Interior of a Hospital Tent, 1918.** Watercolor over pencil on paper; 16 in × 21 in/39 cm × 53 cm. Imperial War Museums, © IWM ART 1611, Lambeth Road, London SE1 6HZ, England, UIK

The generation that endured through what was perhaps the most devastating epidemic ever—the great influenza pandemic of 1918—is now gone. The influenza strain of that pandemic infected about 500 million people, one third of the world’s population, with extraordinarily high pathogenicity and virulence. The result was staggering mortality: an estimated 20 to 100 million lives were lost worldwide. The estimate of deaths of Americans attributable to influenza in that pandemic is 675,000, the majority of which were among those from ages 20 through 40 years. During World War I, the “Great War,” influenza-associated mortality waves occurred in northern Europe, beginning in early summer of 1918 and extending over the course of year; influenza accounted for more fatalities than military engagement. The highest point of combined influenza and pneumonia mortality occurred in October 1918. At the time, the pandemic strain became known as “the Spanish flu,” so called because neutral Spain lacked war censors and was the first country to report on the pandemic publicly; however, the geographic origin of the causative organism remains an enigma. 

Among that generation was the artist John Singer Sargent, who was born in Florence in 1856 and raised principally in France, the child of two Americans: an eye doctor turned medical illustrator father, and an amateur artist mother. Home-schooled and trained at the École des Beaux-Arts in Paris, he enjoyed a storied career, principally as a portraitist. In early summer 1918, late in his renowned career when living in England, Sargent was invited back to France on commission by British Prime Minister Lloyd George, Field Marshal Douglas Haig, and the British Department of Information, War Memorials Committee, to depict the Anglo-American effort in the war. 

During late September, while preparing sketches for his iconic painting *Gassed* in a military camp near Roisel (Péronne), Sargent fell ill with influenza. Sargent was cared for and convalesced in a hospital tent in France. He wrote that he lay there, “with the accompaniment of groans of wounded, and the chokings and coughing of gassed men, which was a nightmare. It always seemed strange on opening one’s eyes to see the level cots and the dimly lit long tent looking so calm, when one was dozing in pandemonium.” 

Sargent’s hospital experience inspired the image featured on this month’s cover, *Interior of a Hospital Tent.* This watercolor depicts the interior of a hospital tent with military cots arrayed in file on the side, covered with blankets in a mix of red (contagious cases) and brown (convalescing or noninfluenza cases), two colors that must have dominated the entire war hospital environment. The scene is actually one of tranquility, a respite from the chaos and carnage of war. In one bed, a soldier lies reading, his head bolstered by pillows; in another, a soldier sleeps on his side with an open tent flap behind him, his bed bathed in light from the world of the healthy. Beyond them, there are three or four more cots with soldiers reclining in varying amounts of darkness and light. Above all, in varying shades of military brown, is a great propped tent canopy.

Sargent remained hospitalized for a week, but unlike so many of the much younger soldiers, he recovered and returned to his task of documenting what he saw. At the outset of his journey through France in July 1918, he had written that the best material for his commission would be to see “a big road encumbered with troop and traffic…combining English and Americans.” By mid-October in northern France, Sargent had had his fill of war. He wrote, “I have wasted lots of time going to the front trenches. There is nothing to paint there—it is ugly, meagre, and cramped…. I have seen what I wanted, roads crammed with troops on the march. It is the finest spectacle that war affords…” 

By the end of October, Sargent returned to Britain to complete the several works for which he had been commissioned. What he had seen firsthand and documented from his experiences in the conflict were the amplification mechanisms for infectious disease transmission that war provides: crowding, migration, and poor ventilation and sanitation. Because there were no vaccines available with proven safety and efficacy to protect against influenza and no antibiotics to treat secondary bacterial infections from influenza or wounds, there were few public health measures available to counter the spread and devastation of the pandemic. 

Whether the great pandemic tipped the balance of power toward the cause of the Allies, such that surrender came in November 1918, remains a matter of debate. The theory that one conflict had a significant impact on the outcome of the other is supported by data published from archives in Austria (Price-Smith 2008), which indicate that waves of morbidity and mortality from influenza were experienced both to a larger extent and earlier among the Central Powers (Germany, Austria-Hungary, the Ottoman Empire, and Bulgaria) than among the Allies. Unfortunately, the pandemic was not limited in its geographic reach, and through 1920, it exerted a tremendous toll on morbidity and mortality and created economic and social burdens, both elsewhere in Europe and throughout the Americas, Africa, Asia, Australia, and the Pacific.

## References

[1] Barry JM. The great influenza: the epic story of the deadliest plague in history. New York: Penguin; 2004.

[2] Bristow NK. American pandemic: the lost worlds of the 1918 epidemic. New York: Oxford University Press; 2012.

[3] Centers for Disease Control and Prevention. History of 1918 flu pandemic [cited 2018 Jun 18]. https://www.cdc.gov/flu/pandemic-resources/1918-commemoration/1918-pandemic-history.htm

[4] Charteris E. John Sargent. Sevenoaks (UK): Pickle Partners Publishing; 2016.

[5] Fairbrother T. John Singer Sargent. New York: Harry N. Abrams; 1994.

[6] Fisher JE. Envisioning disease, gender, and war: women’s narratives of the 1918 influenza pandemic. New York: Palgrave Macmillan; 2012.

[7] Olson S. John Singer Sargent: his portrait. New York: St. Martin’s Press; 1986.

[8] Philips H, Killingray D, editors. The Spanish influenza pandemic of 1918–19: new perspectives. New York: Routledge; 2003.

[9] Price-Smith AT. Contagion and chaos. Cambridge (MA): MIT Press; 2008.

[10] Taubenberger JK, Morens DM. 1918 Influenza: the mother of all pandemics. Emerg Infect Dis. 2006;12:15–22. 10.3201/eid1209.05-0979 16494711 PMC3291398

